# Maternal personality traits moderate treatment response in the Multimodal Treatment Study of attention-deficit/hyperactivity disorder

**DOI:** 10.1007/s00787-019-01460-z

**Published:** 2019-12-20

**Authors:** Guillermo Perez Algorta, Heather A. MacPherson, L. Eugene Arnold, Stephen P. Hinshaw, Lily Hechtman, Margaret H. Sibley, Elizabeth B. Owens

**Affiliations:** 1 LA14YG, Lancaster University, Furness C73, Lancaster, UK; 2 Brown University, Providence, RI USA; 3 The Ohio State University, Columbus, OH USA; 4 University of California, Berkeley, CA USA; 5 McGill University, Montreal, Canada; 6 University of Washington, Seattle, WA USA

**Keywords:** Attention-deficit/hyperactivity disorder, Maternal personality traits, Neuroticism, Conscientiousness, Treatment moderator

## Abstract

Some mothers of children with attention-deficit/hyperactivity disorder (ADHD) present with maladaptive personality profiles (high neuroticism, low conscientiousness). The moderating effect of maternal personality traits on treatment outcomes for childhood ADHD has not been examined. We evaluate whether maternal neuroticism and conscientiousness moderated response in the Multimodal Treatment Study of Children with ADHD. This is one of the first studies of this type. In a randomized controlled trial (RCT), 579 children aged 7–10 (*M* = 8.5); 19.7% female; 60.8% White with combined-type ADHD were randomly assigned to systematic medication management (MedMgt) alone, comprehensive multicomponent behavioral treatment (Beh), their combination (Comb), or community comparison treatment-as-usual (CC). Latent class analysis and linear mixed effects models included 437 children whose biological mothers completed the NEO Five-Factor Inventory at baseline. A 3-class solution demonstrated best fit for the NEO: MN&MC = moderate neuroticism and conscientiousness (*n* = 284); HN&LC = high neuroticism, low conscientiousness (*n* = 83); LN&HC = low neuroticism, high conscientiousness (*n* = 70). Per parent-reported symptoms, children of mothers with HN&LC*,* but not LN&HC, had a significantly better response to Beh than to CC; children of mothers with MN&MC and LN&HC*,* but not HN&LC*,* responded better to Comb&MedMgt than to Beh&CC. Per teacher-reported symptoms, children of mothers with HN&LC*,* but not LN&HC*,* responded significantly better to Comb than to MedMgt. Children of mothers with high neuroticism and low conscientiousness benefited more from behavioral treatments (Beh vs. CC; Comb vs. MedMgt) than other children. Evaluation of maternal personality may aid in treatment selection for children with ADHD, though additional research on this topic is needed.

## Introduction

Despite strengths of existing evidence-based pharmacological and psychosocial treatments for attention-deficit/hyperactivity disorder (ADHD) and disruptive behavior disorders, a substantial number of children fail to respond adequately. Approximately, one-third of children receiving well-managed FDA-approved medication for ADHD do not fully benefit even when augmented with behavioral treatment [1]. In addition, those who demonstrate good initial response may not show sustained benefit beyond 2 years [2, 3]. Thus, enhanced understanding of the relative efficacy of interventions for ADHD and disruptive behavior disorders is needed.

Family preferences, characteristics, and resources can influence the effects of interventions [4–7]. For example, some families may refuse medications or struggle to manage the demands of behavioral treatments amidst a chaotic home environment, psychosocial stressors, or parental psychopathology [6, 8–10]. Therefore, a “one size fits all” approach to treatment may lead to a mismatch between families and interventions [11, 12]. Attending to child and family characteristics in treatment selection may improve long-term outcomes [13], which at this point are suboptimal for many.

The identification of treatment moderators may offer one means of intervention tailoring and matching, by identifying subgroups for whom particular treatments are more or less effective [5, 14, 15]. Such an approach may ultimately enhance the relative efficacy of selected treatments for specific patient populations and facilitate the allocation of limited resources, thereby optimizing outcomes for children and families.

For instance, parental cognitions, attributions, and perceptions have been found to play a role in the therapist–caregiver relationship and influence treatment engagement, compliance, and decisions about continuation vs. termination of services [16–18]. Parental psychosocial factors and mental health issues may be particularly relevant to treatments for disruptive behavior disorders and ADHD in children, which require substantial parent engagement and oversight. For example, in randomized controlled trials (RCTs) of interventions for disruptive behavior disorders in children, low marital adjustment, higher maternal depression, lower familial social class, presence of paternal substance use, and single-mother households were associated with enhanced response to evidence-based psychosocial treatments with parent- and/or child-focused components [5]. In addition, parent training was least effective for economically disadvantaged families, though these families seemed to benefit more from individual vs. group delivery format [8]. For adolescents with conduct problems, Multisystemic Therapy was more effective when fathers were involved in treatment [9]. Thus, parent/family psychosocial and psychopathological factors play an important role in the relative efficacy of interventions for disruptive behavior disorders [5, 8, 9].

Such parent/family variables are also relevant in the treatment of childhood ADHD. For example, high parental anxiety/depression predicted poor response to behavioral interventions in one RCT [6]. In the Multimodal Treatment Study of ADHD (MTA), higher parental education was associated with enhanced response of children’s ADHD symptoms to combination treatment (Comb—medication management and multicomponent behavioral treatment); children from blue-collar and lower SES homes similarly benefited most from Comb in terms of oppositional–aggressive symptoms [10]. Also in the MTA, parental depressive symptoms were associated with worse response to MedMgt, but not Comb [7]. The lack of behavioral support for parents in the MedMgt group may explain the apparent discrepancy with findings reported in Beauchaine et al. [5], who found maternal depression associated with a better response to behavioral intervention. In addition, ethnicity moderated outcomes in the MTA, such that ethnic minority families (African American, Latino) benefitted most from Comb [19]. Although exceptions to these findings exist [20], parental psychosocial factors and psychiatric symptoms are implicated in the phenomenology and treatment response among children with ADHD [6, 7, 10, 19].

Maladaptive parental personality traits may also play a role in response to childhood ADHD treatments. Certain maladaptive personality dimensions have been found to both predict and moderate treatment response for childhood problems other than ADHD. One study found that elevated Cluster B personality disorder (antisocial, borderline, narcissistic, histrionic) symptoms in parents predicted poor response across treatment conditions in a RCT for children with depressive and bipolar disorders [21]. In a RCT of the Oregon model of Parent Management Training for conduct problems, parent antisocial characteristics moderated the effect on coercive but not positive parenting practices [22]. Thus, although not as commonly studied, parental personality traits appear to be implicated in intervention response for childhood psychiatric problems.

Indeed, it is well known that personality traits affect parenting practices. Maladaptive personality profiles, such as high levels of neuroticism (anxious or nervous; prone to stress, guilt, frustration and anger) and low levels of conscientiousness (reliable, rule-abiding, organized, achievement driven) are associated with ineffective parenting (e.g., high levels of negative affectivity toward children) [23–29]. Research has also documented an association between personality traits and coping styles, with conscientiousness predicting adaptive coping (e.g., problem solving), and neuroticism predicting maladaptive coping (e.g., wishful thinking) [30]. Thus, high maternal neuroticism and low maternal conscientiousness in particular may perpetuate problematic functioning in relation to both the individual and family, with potentially important implications for children’s treatment outcomes.

In sum, parental personality traits may be important to consider in treatment selection for childhood ADHD. Indeed, prior research suggests that mothers of children with ADHD present with high levels of neuroticism and low levels of conscientiousness and agreeableness [31–33]. In addition, the idea of a core temperamental propensity to ADHD is corroborated by inattention–disorganization in young adults with low levels of conscientiousness and high neuroticism [34].

The current study examined the moderating effect of maternal personality traits on treatment response in the MTA. Based on prior literature documenting high neuroticism and low conscientiousness in mothers of children with ADHD [31–33], we focused on these personality dimensions. Individuals with high neuroticism present with increased anxiety or nervousness, and are prone to stress, guilt, frustration, and anger, while individuals who are highly conscientious tend to be reliable, rule-abiding, and achievement-driven [25, 35]. We expected these traits to moderate treatment response, given their association with a broad range of variables that could influence the efficacy of interventions for children with ADHD.

We hypothesized the following: first, children of mothers with high levels of neuroticism and low levels of conscientiousness would demonstrate better response to treatments with structured behavioral components (e.g., Beh—intensive behavioral treatment) than those without such structured components (e.g., CC—community comparison treatment-as-usual), because these treatments specifically target parenting practices that may be problematic for mothers with this personality profile. Second, we expected that children with mothers possessing the opposite personality profile (low neuroticism and high conscientiousness) would demonstrate better response to medication (MedMgt) vs. Beh and CC, as these parents may already possess adequate parenting/coping skills, not requiring added supports.

## Methods

### Sample and procedures

This study concerns a secondary analysis of the MTA [36–38]. The sample in the original study included 579 children aged 7–10 (*M* = 8.5, *SD* = 0.8; 19.7% female; 60.8% White) meeting DSM-IV diagnostic criteria for ADHD, combined type. Across six sites, children were randomly assigned in equal proportions to medication management (MedMgt), intensive behavioral treatment (Beh), their combination (Comb), or community comparison treatment-as-usual (CC) for 14 months. MedMgt consisted of a 1-month double-blind titration with methylphenidate [progressing to an open titration with other medications (e.g., d-amphetamine, pemoline, imipramine) if methylphenidate was not better than placebo], followed by maintenance at the optimal dose. Beh consisted of intense, multicomponent individual and group parent training; teacher consultation; a child-focused 8-week full-time summer treatment program with morning academic work and afternoon sports and social skills; and a 12-week half-time classroom behavioral specialist (a summer treatment counselor) to integrate the summer gains into the classroom. Comb integrated the MedMgt and Beh strategies. CC consisted of community treatment of the parents’ choosing; 2/3 obtained medication similar to that given by the MTA but not as carefully managed and at lower doses. Baseline demographic and clinical characteristics across treatment conditions were generally negligible and non-significant.

Participants for the current secondary analysis included 437 children whose biological mothers completed the NEO Five-Factor Inventory [39].

Study procedures were overseen by each site’s institutional review board, and written informed consent and assent were obtained from parents and children, respectively. Additional details about sampling and procedures in the MTA are described elsewhere [36, 37].

### Measures

A comprehensive description of all assessments in the MTA has been outlined previously [40].

Children’s ADHD and disruptive behavior symptom severity was measured at baseline and 3, 9, and 14 months via the parent- and the teacher-reported Swanson, Nolan, and Pelham (SNAP) rating scale [41]. The SNAP includes inattention and hyperactivity/impulsivity subscales and has good internal consistency, inter-rater reliability (between teachers and parents), and predictive validity [41, 42]. It also included ratings of oppositional–defiant disorder (ODD) symptoms on the same 0–3 metric. Because the MTA behavioral treatment addressed both ADHD and disruptive behavior symptoms, we included in our outcome variable both the 18 ADHD and the 8 ODD symptoms, using the SNAP item mean. Children’s diagnostic status (ODD, anxiety) were determined through formal initial diagnosis using DISC-IIIR.

Biological mothers’ mental health status was determined through report of primary informant (89% of informants were biological mothers) at study entry. Questions assessed whether the biological mother had mental health and nervous problems and type of treatment received, if any.

Biological mothers’ personality traits were measured at 3 months via the NEO Five-Factor Inventory [39]. This study focused specifically on neuroticism and conscientiousness. Example items are: (1) neuroticism, “I often feel tense or jittery,” and (2) conscientiousness, “When I make a commitment, I can always be counted on to follow through.” The NEO Five-Factor Inventory has strong psychometric properties [43].

Biological mothers’ depressive symptoms were measured at baseline via the Beck Depression Inventory (BDI) [44]. The BDI internal consistency, test–retest reliability, and convergent validity have been adequately demonstrated [44]. Maternal depression was included, because a previous qualitative ROC analysis [7] found it to be a significant moderator of treatment response in this sample. Biological mothers’ ADHD-related symptom dimensions were measured at baseline via the Conners’ Adult ADHD Rating Scales (CAARS) [45]. The CAARS has good internal reliability, test–retest reliability, sensitivity, and specificity [46]. Consistent with other MTA analyses, empirically derived factors of inattention/cognitive problems, hyperactivity/restlessness, and impulsivity/emotional lability were examined [47].

Negative/ineffective discipline was measured using Hinshaw et al. [48] second order factor (Chronbach *α* = 0.83), derived from the Alabama Parenting Questionnaire and the Parent Child Relationship Questionnaire first order factors.

### Statistical analyses

First, latent class analysis (LCA via Mplus Version 8.1) was used to estimate subgroups of mothers with different combinations of NEO neuroticism and conscientiousness scale levels. LCA identifies latent subgroups of mothers using their pattern of responses to NEO subscales [49, 50]. BIC, entropy, parsimony, and substantive interpretability guided model selection [50].

Second, we evaluated if there were differences in demographics, treatment allocation, and child and maternal clinical characteristics between latent classes. For example, we tested if there were differences in the number of children with comorbid anxiety disorder, a factor that has been shown to predict treatment responses in previous studies [5, 51]. We also evaluated if there were differences in mothers’ ADHD-related symptom dimensions and negative/ineffective discipline between classes. We conducted these analyses in light of the idea that personality traits affect parenting practices, and because of the possibility of having a core temperamental propensity to ADHD related with a certain combination of personality traits [35].

Third, linear mixed effects modeling (LME via SAS version 9.4) was used to test the moderating effect of the resulting maternal personality latent classes on children’s ADHD symptom severity, using longitudinal SNAP measurements at baseline, 3, 9 and 14 months. Models included fixed effects for time (log of the number of days since randomization in MTA [38]), treatment, and NEO latent classes; children’s ADHD symptom severity (measured via parent- and teacher-reported SNAP) as the dependent variable; and a subject-specific random intercept and slope. Moderation was tested using the three-way interaction of time × NEO latent class × treatment, with treatment operationalized using a set of previously developed orthogonal contrasts: (1) MedMgt&Comb vs. CC&Beh; (2) MedMgt vs. Comb; (3) and Beh vs. CC [2, 52]. For NEO latent class, we generated dummy variables. As a sensitivity analysis, we expanded the previous model incorporating maternal depression and self-report history of mental/nervous problems as covariates.

## Results

### Latent class analysis

Comparing the fit of latent class models for 1–4 classes, a 3-class solution demonstrated best fit for neuroticism and conscientiousness, with a satisfactory proportion of mothers per class (Table 1).

**Table 1:** Model fit results for latent classes of different levels of neuroticism and conscientiousness in biological mothers of children with attention-deficit/hyperactivity disorder

	1 class	2 classes	3 classes	4 classes
Free parameter	4	7	10	13
AIC	5986.938	5904.854	5877.001	5876.730
BIC	6003.258	5933.413	5917.801	5929.769
Sample-adjusted BIC	5990.564	5911.199	5886.066	5888.514
Entropy	N/A	.62	.70	.70
Log-likelihood value		− 2989.469^§^	− 2945.427^§§^	− 2928.501
*N* for each class		C1 = 123 C2 = 314	C1 = 284 C2 = 83 C3 = 70	C1 = 16 C2 = 279 C3 = 70 C4 = 72

*AIC *Akaike information criterion, *BIC *Bayesian information criterion

^§^Vuong–Lo–Mendell–Rubin likelihood ratio test for 1 (H0) vs. 2 classes, *p* < .05

^§§^Vuong–Lo–Mendell–Rubin likelihood ratio test for 2 (H0) vs. 3 classes, *p* < .05

**Fig. 1:**
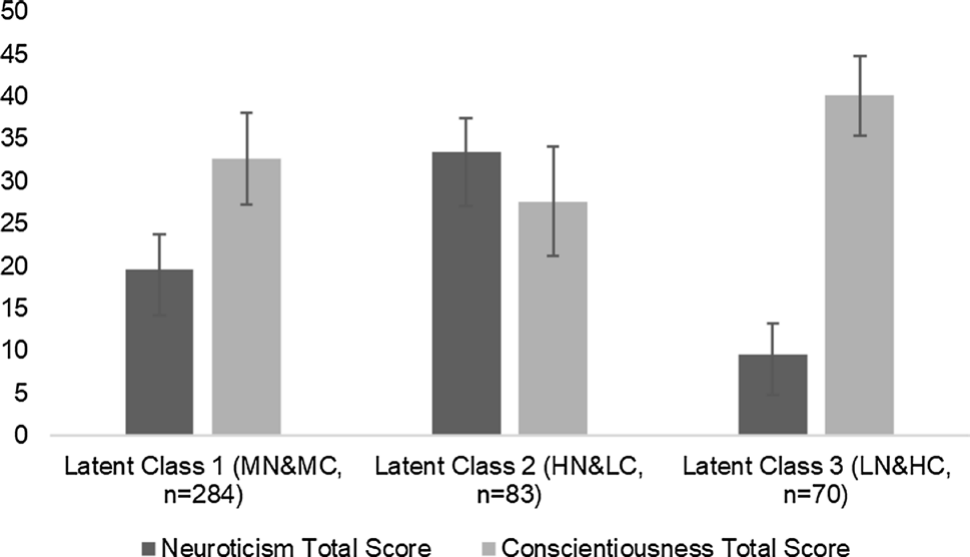
Latent classes of biological mothers with different levels of neuroticism and conscientiousness and error bars. *MN&MC *moderate neuroticism and moderate conscientiousness, *HN&LC *high neuroticism and low conscientiousness, *LN&HC *low neuroticism and high conscientiousness

Figure 1 displays the three NEO neuroticism and conscientiousness classes. Mothers in the first class had moderate levels of neuroticism and conscientiousness (MN&MC, *n* = 284, 65%). Mothers in the second class had a high level of neuroticism and a low level of conscientiousness (HN&LC*, n* = 83, 19%). Mothers in the third class had a low level of neuroticism and a high level of conscientiousness (LN&HC*, n* = 70, 16%).

Table 2 presents demographic, clinical information, and treatment allocation for children of mothers in the empirically derived classes. There were significant differences in depression and ADHD-related symptom dimensions, such that mothers in the HN&LC class reported a greater number of depressive and ADHD symptoms than mothers in other classes. The HN&LC class also self-reported significantly more mental/nervous problems at baseline than mothers in the MN&MC and LN&HC classes. The HN&LC (and MN&MC) classes also showed significantly higher scores of negative/ineffective discipline than LN&HC*.*

**Table 2:** Demographic, clinical characteristics and treatment allocation of latent classes of biological mothers with different levels of neuroticism and conscientiousness

		Latent class 1 MN&MC (*n* = 283), 65%	Latent class 2 HN&LC (*n* = 80), 19%	Latent class 3 LN&HC (*n* = 67), 16%	χ^2^ or *F*
Child age at baseline, M (SD)		7.8 (0.8)	7.8 (0.8)	7.7 (0.7)	1.22
Child any anxiety disorder, *n *(%)^φ^		107 (39)	37 (46)	21 (32)	2.97
Child oppositional–defiant disorder, *n *(%)^φφ^		119 (44)	29 (38)	28 (42)	1.00
Mother’s age when child was born, M (SD)^#^		27.8 (5.9)	28.4 (6.2)	29 (5.4)	1.21
Mother’s age when completing NEO, M (SD)		35.1 (7.5)	36.5 (6.1)	35.6 (8)	1.08
Married, *n *(%)		200 (71)	49 (61)	40 (60)	4.54
High school education (or greater), *n *(%)		270 (95)	76 (95)	65 (97)	.41
Full-time job, *n *(%)		150 (53)	41 (51)	42 (63)	2.39
Neuroticism, M (SD)		16.69 (4.06)^a^	33.55 (3.85)	9.52 (3.75)	689.5***
Conscientiousness, M (SD)		32.72 (5.44)^a^	27.65 (6.5)	40.1 (4.66)	93.43***
Negative/ineffective discipline, M (SD)^##^		.77 (1.56)	1.20 (1.48)	.07 (1.61)^b^	9.65***
BDI, M (SD)^###^		.28 (.24)	.58 (.36)	.13 (.14)	65.4***
CAARS inattention/cognitive problems, M (SD)^####^		.67 (.51) ^d^	1.14 (.61)^c^	.34 (.35)	42.20***
CAARS hyperactivity/restlessness, M (SD)		.77 (.55)	.95 (.52)^c^	.60 (.52)	6.85**
CAARS impulsivity/emotional lability, M (SD)		.64 (.39) ^d^	1.05 (.41)^c^	.37 (.31)	53.71***
Mental/nervous problems, *n *(%)^#####^		47 (18)	30 (40)	4 (7)	25.1***
Combination (Comb), *n *(%)		69 (24)	18 (23)	21 (31)	
Medication management (MedMgt), *n *(%)		59 (21)	24 (30)	15 (23)	
Intensive behavioral treatment (Beh), *n *(%)		70 (25)	22 (27)	19 (28)	
Community comparison treatment (CC), *n *(%)		85 (30)	16 (20)	12 (18)	

*MN&MC *moderate neuroticism and moderate conscientiousness, *HN&LC *high neuroticism and low conscientiousness, *LN&HC *low neuroticism and high conscientiousness, *BDI *Beck Depression Inventory, *CAARS *Conners’ Adult ADHD Rating Scales

*p < .05; **p < .01; ***p < .001

^a^Games–Howell post hoc test shows that all pairwise comparison between classes are statistically significantly different (*ps* < .0001)

^b^Games-Howell post hoc test shows that LN&HC class score was significantly lower than MN&MC and HN&LC

^c^Games-Howell post hoc test shows that HN&LC class score was significantly higher than MN&MC and LN&HC

^d^MN&MC class score was significantly higher than LN&HC

^φ^Sample with complete data: 275, 80 and 65, respectively

^φφ^Sample with complete data: 270, 77 and 66, respectively

^#^Sample with complete data: 274, 80 and 64, respectively

^##^Sample with complete data: 278, 80 and 66, respectively

^###^Sample with complete data: 280, 80 and 66, respectively

^####^Sample with complete data: 254, 73 and 58, respectively

^####^Sample with complete data: 259, 75 and 59, respectively

### Moderation analysis

#### Parent report

**Fig. 2:**
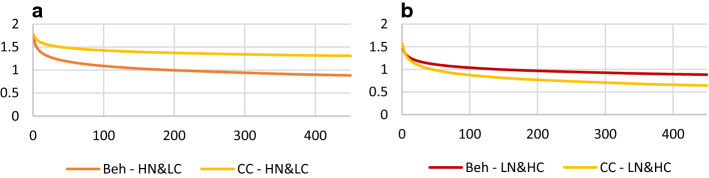
Maternal neuroticism and conscientiousness as moderators of treatment response. **a** Shows how children of mothers in HN&LC class receiving Beh (red line) demonstrated a better treatment response than children with mothers in the same class who received CC (orange line). **b** Shows no difference in children’s treatment response to Beh or CC with mothers in the LN&HC. *HN&LC *high neuroticism and low conscientiousness, *LN&HC *low neuroticism and high conscientiousness, *Beh *behavioral, *CC *community comparison

Two significant three-way interactions emerged regarding SNAP parent report. The largest effect occurred when comparing the treatment response between Beh and CC (*b* = − 0.12, se = 0.05, *p* = 0.009). Children of mothers in the HN&LC class who received Beh demonstrated a significantly better response (simple slope *b* = − 0.31, se = 0.04, CI = − 0.39 to − 0.24) than children with mothers in the same class who received CC (simple slope *b* = − 0.18, se = 0.04, CI = − 0.26 to − 0.11) (Fig. 2a). In contrast, children with mothers in the LN&HC class had a non-significant differential treatment response in the opposite direction: (Beh simple slope *b* = − 0.24, se = 0.04, CI = − 0.31 to − 0.16 and CC simple slope *b* = − 0.35, se = 0.05, CI = − 0.45 to − 0.26) (Table 3; Fig. 2).

**Table 3:** Maternal neuroticism and conscientiousness as moderators of treatment response

	SNAP parent report	SNAP teacher report
	*b* (se) [95% CI]	*b* (se) [95% CI]
Intercept	1.54 (.05) [1.43; 1.66]	1.82 (.07) [1.69; 1.95]
Time	− .29 (.02) [− .34; − .25]	− .35 (.03) [− .42; − .29]
MN&MC	.14 (.07) [.009; .27]	− .05 (.07) [− .19; .10]
HN&LC	.21 (.08) [.05; .37]	− .10 (.09) [− .28; .08]
Comb&MedMgt vs. Beh&CC	− .08 (.06) [− .19; .04]	− .08 (.07) [− .21; .05]
Comb vs. MedMgt	− .08 (.08) [− .24; .07]	− .14 (.09) [− .32; .04]
Beh vs. CC	− .04 (.09) [− .21; .14]	.08 (.10) [− .12; .28]
Time × Comb&MedMgt vs. Beh&CC	− .05 (.02) [− .10; − .006]	− .07 (.03) [− .13; − .008]
Time × Comb vs. MedMgt	.03 (.03) [− .04; .09]	.04 (.04) [− .04; .12]
Time × Beh vs. CC	.06 (.04) [− .01; .13]	− .01 (.04) [− .10; .08]
Time × MN&MC	.04 (.03) [− .02; .09]	.04 (.03) [− .03; .11]
MN&MC × Comb&MedMgt vs. Beh&CC	.14 (.07) [.01; .27]	.06 (.07) [− .09; .21]
MN&MC × Comb vs. MedMgt	.08 (.09) [− .10; .25]	.04 (.10) [− .16; .24]
MN&MC × Beh vs. CC	− .01 (.09) [− .20; .18]	− .07 (.11) [− .29; .14]
Time × HN&LC	.05 (.03) [− .01; .11]	.05 (.04) [− .03; .13]
HN&LC × Comb&MedMgt vs. Beh&CC	.05 (.08) [− .11; .21]	− .003 (.09) [− .18; .17]
HN&LC × Comb vs. MedMgt	.18 (.11) [− .03; .39]	.26 (.12) [.02; .50]
HN&LC × Beh vs. CC	.12 (.12) [− .12; .35]	− .03 (.13) [− 29; .23]
Time × MN&MC × Comb&MedMgt vs. Beh&CC	**−** **.05 (.03) [−** **.11; -.001]**	− .01 (.03) [− .08; .06]
Time × MN&MC × Comb vs. MedMgt	− .03 (.04) [− .10; .04]	− .01 (.04) [− .10; .08]
Time × MN&MC × Beh vs. CC	− .04 (.04) [− .12; .03]	.001 (.05) [− .09; .10]
Time × HN&LC × Comb&MedMgt vs. Beh&CC	− .01 (.03) [− .07; .05]	.003 (.04) [− .08; .08]
Time × HN&LC × Comb vs. MedMgt	− .07 (.04) [− .16; .01]	**−** **.13 (.06) [−** **.24; −** **.02]**
Time × HN&LC × Beh vs. CC	**−** **.13 (.05) [−** **.22; −** **.03]**	− .02 (.06) [− .14; .10]

LN&HC was the reference class in hierarchical linear models

*SNAP *Swanson, Nolan, And Pelham ADHD Rating Scale, *MN&MC *moderate neuroticism and moderate conscientiousness, *HN&LC *high neuroticism and low conscientiousness, *Comb *combined, *MedMgt *medication management, *Beh *Behavioral, *CC *community comparison

**Fig. 3:**
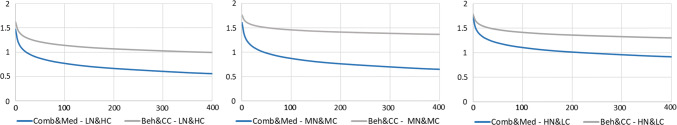
Maternal neuroticism and conscientiousness as moderators of treatment response. *LN&HC *low neuroticism and high conscientiousness, *MN&MC *moderate neuroticism and moderate conscientiousness, *HN&LC *high neuroticism and low conscientiousness, *Comb *combined, *MedMgt *medication management, *Beh *behavioral, *CC *community comparison. The three graphs above show a decreasing improvement with Comb&MdMgt (systematic medication, blue line) as you go from LN&HC to HN&LC (left to right). LN&HC did better with Beh&CC (gray line) than MN&MC, while HN&LC did less well with Comb&MedMgt, the two arms with systematic medication

The other significant effect on parent-rated symptoms (*b* = − 0.05, se = 0.03, *p* = 0.04) reflected the main MTA outcome finding for the bulk of the sample [38] for all but the HN&LC group. That is, Comb&MedMgt was better than Beh&CC for MN&MC and LN&HC, but not for HN&LC who did better with Beh&CC (Fig. 3)*.* Children with mothers in the MN&MC class (over half of the sample) who received Comb&MedMgt demonstrated better treatment response (simple slope *b* = − 0.37, se = 0.02, CI = − 0.40 to − 0.33) than children with mothers in the same class who received Beh&CC (simple slope *b* = − 0.15, se = 0.02, CI = − 0.18 to − 0.12). Similarly, children with mothers in the LN&HC class responded better to Comb&MedMgt (simple slope *b* = − 0.35, se = 0.03, CI = − 0.41 to − 0.29) than to Beh&CC (simple slope *b* = − 0.24, se = 0.04, CI = − 0.31 to − 0.17).

#### Teacher report

**Fig. 4:**
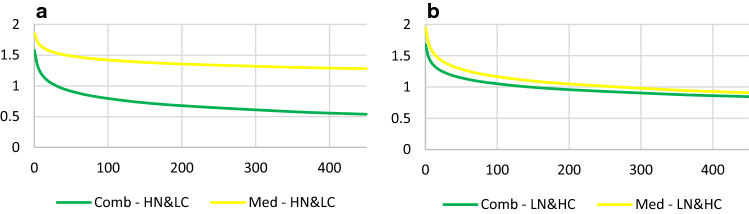
Maternal neuroticism and conscientiousness as moderators of treatment response. **a** Shows how children of mothers in HN&LC class receiving Combined (green line) demonstrated a better treatment response than children with mothers in the same class who received MedMgt (yellow line). **b** Shows no difference in children’s treatment response to Comb or MedMgt with mothers in the LN&HC. *HN&LC *high neuroticism and low conscientiousness, *LN&HC *low neuroticism and high conscientiousness, *Comb *combined, *MedMgt *medication management

One significant three-way interaction emerged for SNAP teacher report (*b* = − 0.13, se = 0.06, *p* = 0.02). Children with mothers in the HN&LC class who received Comb demonstrated better treatment response (simple slope *b* = − 0.39, se = 0.05, CI = − 0.49 to − 0.30) than children with mothers in the same class who received MedMgt (simple slope *b* = − 0.22, se = 0.05, CI = − 0.31 to − 0.13). The difference between these two treatments was non-significant in the opposite direction for children with mothers in the LN&HC class (Comb simple slope *b* = − 0.31, se = 0.05, CI = − 0.41 to − 0.22; MedMgt simple slope *b* = − 0.40, se = 0.06, CI = − 0.48 to − 0.30) (Table 3; Fig. 4).

### Sensitivity analysis

Two sensitivity analysis models evaluated the robustness of aforementioned findings, by covarying maternal depression and self-report history of mental/nervous problems. When using SNAP parent report as the dependent variable (*n* = 393), the time × HN&LC × Beh vs. CC and time × MN&MC × Comb&MedMgt vs. Beh&CC interactions remained significant (*b* = − 0.11, se = 05, *p* = 0.03 and *b* = − 0.06, se = 03, *p* = 0.02, respectively). Additionally, the time × HN&LC × Comb vs. MedMgt interaction emerged as significant in this model (*b* = − 0.10, se = 05, *p* = 0.03). When using SNAP teacher report as the dependent variable (*n* = 392), time × HN&LC × Comb vs. MedMgt interaction remained the sole significant three-way interaction (*b* = − 0.13, se = 06, *p* = 0.03), as in the original model.

## Discussion

This is one of the first studies to examine the moderating effect of maternal personality traits on treatment response for children with ADHD. Mothers presented with different levels of neuroticism and conscientiousness, and mothers with high neuroticism and low conscientiousness (*n* = 83/437) reported greater depressive symptoms, ADHD symptoms, and mental/nervous problems than mothers with moderate or low neuroticism and moderate or high conscientiousness. Moreover, levels of neuroticism and conscientiousness moderated treatment effects. Specifically, children with mothers in the HN&LC class who received Beh demonstrated better response on SNAP parent report than children with mothers in the same class who received CC, whereas treatment response trended (nonsignificantly) in the opposite direction for children with mothers in the LN&HC class. Comparing treatments involving medication, Comb&MedMgt were predictably better than Beh&CC for children with mothers in the MN&MC or LN&HC class, as was the case for the whole MTA sample [1, 36]. However, this well-established MTA finding was not found for the HN&LC class. Finally, for SNAP teacher report, children with mothers in the HN&LC class who received Comb demonstrated better treatment response than children with mothers in the same class who received MedMgt, whereas the difference between the two treatments was actually nonsignificant in the opposite direction for children with LN&HC mothers. Importantly, results remained significant after adjusting for maternal depression and mental/nervous problems. These findings for both parent- and teacher-rated ADHD and oppositional–defiant symptom severity paints a picture of maternal high neuroticism and low conscientiousness predicting a better response to behavioral treatment and relatively lesser response to medication.

The finding that mothers in the HN&LC class reported higher depression, and ADHD symptoms measured with the CAARS, mental/nervous problems, and negative/ineffective discipline is in line with prior research documenting that parents of children with ADHD present with high rates of depression, ADHD symptoms, and parenting stress [31, 53, 54], in addition to increased neuroticism and decreased conscientiousness [31–33].

In terms of moderator findings, the effect on teacher ratings suggests a real moderation of treatment response, not just an effect of parent personality on parent ratings. Collectively, results suggest that children of mothers with high neuroticism and low conscientiousness may benefit most from structured behavioral treatments, either alone or added to medication. High neuroticism and low conscientiousness are associated with poor coping [30] and ineffective parenting practices [23–29]. Because behavioral treatments specifically target these deficits, children of personality-impaired mothers may demonstrate the largest gains in response to newly implemented effective parenting strategies. In contrast, mothers with more adaptive personality profiles (low neuroticism and high conscientiousness) may have enjoyed effective parenting and coping abilities from the start, and thus did not benefit as much from targeted, structured interventions for these skills. Indeed, improvements in negative/ineffective parental discipline mediated outcomes in the MTA [48]. Insofar as anxious, neurotic mothers tend to have anxious children, the finding that children of HN&LC mothers respond relatively better to behavioral treatment than children of LN&HC mothers is consistent with prior MTA findings documenting that children with comorbid anxiety responded relatively better to behavioral treatment [38]. In addition, these results are consistent with structured evidence-based treatments tending to be most effective for children and families with the greatest impairment [21, 22]. Thus, parental assessment prior to treatment initiation for childhood mental health problems, including ADHD, may help inform the most effective and optimal interventions.

An alternate possible explanation arises from the intensive and multicomponent nature of the MTA behavioral intervention, incorporating not only individual and group parent training, but also teacher consultation, a child-directed 8-week full-time summer treatment program, and a 12-week half-time classroom paraprofessional behavioral aide. It is therefore possible that the children of more impaired mothers benefitted to a greater extent from the consistency and support of extensive “wrap-around” services they received and not from parental changes per se. Further research is required to determine the mechanism through which behavioral treatments and maternal personality traits interact to facilitate improvement in children’s ADHD symptoms.

Findings should also be viewed within the context of another significant parental moderator of MTA treatment response: parental depression. Specifically, baseline parental depressive symptoms were associated with worse response in MedMgt and Comb [7]. In these analyses, Owens et al. suggested that parental depressive symptoms may have interfered with children’s receipt of medication (inconsistency with doctor visits, filling prescriptions, administering medications), therefore thwarting the potential therapeutic effect of such pharmacologic intervention. In contrast, because parents received added supports and training via behavioral treatment, this was thought to have mitigated the negative effect of parental depression on children’s ADHD symptoms [7]. These prior findings are in line with current results, which similarly suggest that behavioral interventions may be most effective for mothers with coping problems. Importantly, because personality traits represent more stable and enduring deficits than waxing/waning psychopathology (such as depression), personality may be more useful to measure and consider when selecting treatment. Further research is needed to clarify the relation between maternal personality traits, depression, and children’s ADHD symptoms, as depressive symptoms may mediate the relationship between maternal personality and treatment response, or vice versa.

It is interesting that the superiority of behavioral treatment for children of HN&LC mothers relative to children of LN&HC mothers was manifested in different orthogonal contrasts for parent and teacher ratings. For parent ratings, it manifested as significant superiority of behavioral treatment alone over routine community care, which at that time was mainly suboptimal medication; this was the “behavioral substitution effect”. But for teacher ratings, in the context of medication dramatically impacting classroom performance, it manifests as superiority of combination over medication alone—the “behavioral additive effect”. At the very least, findings highlight convergence of moderation findings between parental depression and maternal personality, though with some nuances.

## Limitations

Despite the strengths of this study, findings should be interpreted within the context of limitations. First, analyses were restricted to personality traits of biological mothers, to reduce potential confounds. However, personality traits of other caregivers may also influence treatment response. Second, these personality traits were measured 3 months after baseline. This is not an optimal situation to test moderators. Third, there was limited variability in conscientiousness vs. neuroticism, which may have limited power to detect significant differences. Families choosing to enroll in an RCT in general may be more compliant and conscientious than families who opt out of RCTs. Fourth, analyses focused on the personality traits of neuroticism and conscientiousness, given prior literature documenting this profile in mothers of children with ADHD [31–33], but other personality dimensions may also influence treatment outcomes such as novelty seeking [55]. Fifth, biological mothers’ mental health status was based on self-report. Sixth, analyses were restricted only to participants whose biological mothers completed the NEO Five-Factor Inventory. Seventh, the generalizability of these findings may be limited by the intensity of the MTA behavioral treatment, which consisted of 35 parent training sessions, 10 teacher consultations, an 8-week-all-day summer treatment program, and 12 weeks of a half-time paraprofessional aid in the classroom. Less comprehensive behavioral programs may not show the same results. Finally, although it is a strength that moderation findings were maintained when adjusting for maternal depression and mental/nervous problems, it is possible that other unmeasured parent variables moderated treatment response or at least partially accounted for the relationship observed in the current study between maternal personality and treatment response. For example, prior research has identified numerous other parental factors that can influence children’s treatment response, including: ethnicity, marital adjustment, social class, education level, family composition, paternal involvement in treatment, internalizing symptoms, substance use, and cluster B personality disorder symptoms [5–10, 19, 21, 22].

Thus, future research should further explore: (1) personality in a variety of different caregivers; (2) if dyadic personality profiles (between two caregivers or between caregiver and child) have a stronger impact on treatment response; (3) other personality dimension; (4) the impact of child comorbid anxiety; and (5) a wider variety of possible parent/family predictors and moderators.

## Clinical implications

Children with ADHD who have mothers with more impaired personality profiles (high neuroticism and low conscientiousness) appear to benefit most from the structured MTA behavioral interventions (by targeting maladaptive parenting and coping via parent training, and/or by extensive wrap-around services including teacher consultation, summer treatment program, and a classroom behavioral aide). In contrast, children of mothers with more adaptive personality profiles (moderate/low neuroticism and moderate/high conscientiousness) may experience adequate improvement in ADHD symptoms with pharmacotherapy alone, because these mothers are likely to be more skilled and/or have less room for growth. Thus, maternal personality could inform treatment planning for childhood ADHD (e.g., more impaired mothers may require behavioral treatments), though additional research is needed and other moderating factors must be considered. Nevertheless, maternal personality profiles may be important targets in future intervention development work.
